# Unraveling the Concealed Transcriptomic Landscape of PTEN in Human Malignancies

**DOI:** 10.2174/0113892029265367231013113304

**Published:** 2023-12-12

**Authors:** Michaela A. Boti, Panagiotis G. Adamopoulos, Dido Vassilacopoulou, Andreas Scorilas

**Affiliations:** 1 Department of Biochemistry and Molecular Biology, Faculty of Biology, National and Kapodistrian University of Athens, Athens, Greece

**Keywords:** Phosphatase and tensin homolog, alternative splicing, long-read sequencing, tumor suppression, splice variants, cytogenetic

## Abstract

**Background:**

Phosphatase and tensin homolog, widely known as PTEN, is a major negative regulator of the PI3K/AKT/mTOR signaling pathway, involved in the regulation of a variety of important cellular processes, including cell proliferation, growth, survival, and metabolism. Since most of the molecules involved in this biological pathway have been described as key regulators in cancer, the study of the corresponding genes at several levels is crucial.

**Objective:**

Although previous studies have elucidated the physiological role of PTEN under normal conditions and its involvement in carcinogenesis and cancer progression, the transcriptional profile of PTEN has been poorly investigated.

**Methods:**

In this study, instead of conducting the “gold-standard” direct RNA sequencing that fails to detect less abundant novel mRNAs due to the decreased sequencing depth, we designed and implemented a multiplexed PTEN-targeted sequencing approach that combined both short- and long-read sequencing.

**Results:**

Our study has highlighted a broad spectrum of previously unknown PTEN mRNA transcripts and assessed their expression patterns in a wide range of human cancer and non-cancer cell lines, shedding light on the involvement of PTEN in cell cycle dysregulation and thus tumor development.

**Conclusion:**

The identification of the described novel PTEN splice variants could have significant implications for understanding PTEN regulation and function, and provide new insights into PTEN biology, opening new avenues for monitoring PTEN-related diseases, including cancer.

## 1 INTRODUCTION

Cytogenetic and molecular studies conducted in the 1980s have unveiled the partial or complete loss of chromosome 10 in breast, prostate, and brain cancers [1]. However, it was not until 1997 that subsequent research works led to the identification of a novel candidate tumor suppressor gene at the 10q23 locus, named phosphatase and tensin homolog (PTEN). These studies have demonstrated that PTEN is frequently disrupted in multiple sporadic tumor types and targeted by germline mutations in patients with cancer predisposition syndromes, such as Cowden disease [2, 3]. Thereupon its identification, PTEN knockouts in mice confirmed its essential tumor suppressive role in multiple tissue types and displayed its significant involvement in embryonic development [4-6]. Interestingly, the study on hypomorphic PTEN mouse models’ series indicated the overwhelming functional consequences of the subtle PTEN downregulation, which benefits cancer susceptibility and favors tumor progression [4, 7, 8]. In addition to genomic perturbations leading to inactivation of a PTEN allele, there is scientific evidence that alterations in expression levels or aberrant subcellular compartmentation of the encoded protein are associated with cancer, underlying the multiple pathways that converge in tumorigenesis [9, 10].

The human PTEN protein is a 403-aa dual phosphatase with both protein and lipid phosphatase activity [11]. Its principal catalytic function is to dephosphorylate phosphatidylinositol3,4,5trisphosphate (PIP3), a potent activator of 3phosphoinositide-dependent kinase (PDK) and AKT12 [12]. Excessive concentration of PIP3 in the plasma membrane after PTEN loss leads to recruitment and hyperactivation of cell membrane-related proteins that regulate cell cycle and proliferation.

Previous studies indicate that PTEN is active as a homodimer, suggesting that yet unknown mechanisms in cancer impair the homodimerization of PTEN, leading to its inactivation [13]. Besides the canonical PTEN isoform, an additional coding variant named PTEN-Long (PTEN-L) has been identified. PTEN-L is a 576-amino acid protein that translates from a non-canonical CTG initiation codon, which is located on exon 1, a few hundred bases upstream of the annotated initiation codon. Compared to the canonical isoform, PTEN-L possesses an additional region of 176 amino acids at the N-terminus, constituting a signal peptide known as PTEN-L leader [14, 15]. The particular leader contains a polyalanine permeability signal and a poly-arginine re-entry stretch, both of which are required for the permeation of PTEN-L through cell membrane barriers. In contrast to the canonical PTEN, PTEN-L is secreted into the extracellular space and re-enters adjacent cells, assuming similar roles as PTEN *in vivo* and *in vitro*. In addition to PTEN-L, another PTEN isoform, named PTENβ, has been identified. Likewise, PTENβ is encoded by an alternative initiation codon at an AUU site in the 5´untranslated region of PTEN transcript v.1, and is thus characterized by the presence of 146 additional amino acids in its N-terminus compared to the main PTEN isoform. Even though PTEN-L and PTENβ share high homology with the main PTEN in terms of their primary structure, it is suggested that these isoforms exhibit additional functions and/or undergo different regulation mechanisms due to their extended N-terminus [16-20]. Interestingly, previous studies have already shown that PTEN-L and PTENβ promote carcinogenesis by directly interacting with WDR5 to promote H3 lysine 4 (H3K4) trimethylation and maintain a tumor-promoting state, confirming PTEN's multifaced role in cancer development and progression [21].

PTEN is a major negative regulator of the PI3K/ AKT/mTOR signaling pathway, controlling a wide range of essential cellular processes, including cell proliferation, growth, survival, and metabolism [12, 22-24]. Considering the biological importance of its function and the profound pathological effects that arise as a consequence of changes in PTEN’s expression levels and/or functionality of the corresponding protein, this vital gene represents a key factor in normal and pathological conditions [25, 26]. Regarding its involvement in cancer, it is known that PTEN is one of the most mutated tumor suppressor genes and is often suppressed or downregulated if it is not mutated [27-30]. Of note, PTEN has different loss patterns in different tumor types, suggesting that the mechanisms leading to dysregulation depend on the tissue involved. From a structural standpoint, crystallization analysis data indicated that PTEN protein is characterized by the presence of four domains. In detail, the N-terminal region of the particular protein encompasses a PIP2-binding domain (PBD) (residues 1-14) and a phosphatase domain (residues 15-185) that contains a catalytic signature motif, HCXXGXXR (X represents any amino acid), also present in the active sites of other protein tyrosine phosphatases (PTPs) [31, 32]. The next PTEN domain, which along with the phosphatase domain compromises the most significant parts of the enzyme, is the C2 domain (residues 186-351). Particularly, the C2 domain is responsible for the binding of the phospholipids of the membrane to the enzyme. The particular domain is crucial for the regulation of this protein, as it is involved in the interaction of PTEN with other proteins that positively or negatively regulate the function of PTEN. Finally, PTEN also has a C-terminal tail (residues 352-401) and a PDZ domain interaction motif (residues 402 and 403), which together with the C2 domain form the C-terminal region of the enzyme.

The transcriptional profile of PTEN remains poorly investigated, which is mainly due to the serious drawbacks of the existing sequencing technologies. Even though the introduction of next-generation sequencing (NGS) has paved the way for the exploration of transcriptome and the research on alternative splicing, leading to the detection of previously uncharacterized mRNA transcripts [33], this technology still involves serious limitations, with its most crucial disadvantage being the generation of short sequencing reads (up to ~600 bp). These restrictions were overcome with the launching of third-generation sequencing (TGS) technologies, such as the one offered by Oxford Nanopore Technologies (ONT). Nanopore sequencing is characterized by improved sequencing chemistry, resulting in the production of long reads, with an average length of more than 10 kb [34, 35]. The long-read technology of Nanopore sequencing is its most advantageous feature, since it has improved the quality of genome assembly and genome analysis, and is expected to facilitate the characterization of novel transcripts, shedding light on the hallmarks of alternative splicing toward human pathophysiology [36].

The present study aimed to characterize novel PTEN mRNA transcripts and evaluate their expression pattern in different tumor types, as well as in non-cancerous cell lines using the state-of-the-art nanopore sequencing technology. Through a targeted sequencing approach that involved the combination of short- and long-read sequencing, leading to highly accurate nanopore sequencing reads, we have unveiled the complex transcriptional profile of PTEN in multiple human cancers as well as in non-cancerous cells, offering new insights into its multifaceted role under normal and pathological conditions.

## 2 MATERIALS AND METHODS

### 2.1 Biological Material

The present work included a wide spectrum of human cell lines that were purchased from the American Type Culture Collection (ATCC), and propagated according to its guidelines. All experiments were performed on mycoplasma-free cells and the utilized cell lines have been authenticated using SNP profiling. The established panel of cell lines that was used is as follows: SH-SY5Y, H4, U-87 MG, U-251 MG (brain cancer), SK-BR-3, BT-20, MCF-7, BT-474 (breast cancer), HeLa, SiHa (cervical carcinoma), DLD-1, HT-29, Caco-2, HCT 116, SW620 (colorectal cancer), HEK-293 (embryonic kidney), 786-O, Caki-1 (renal cell carcinoma), Hep G2, HuH-7 (hepatocellular carcinoma), A549 (lung carcinoma), Raji, SU-DHL-1 (lymphoma), ES-2, SK-OV-3, OVCAR-3, MDAH-2774 (ovarian cancer), 1.2B4 (pancreas), PC-3, DU 145, LNCaP (prostate cancer), HL-60 (leukemia), HaCaT, BJ (Skin), AGS (gastric adenocarcinoma), RT4, T24 (urinary bladder cancer), Ishikawa, and SK-UT-1B (endometrial adenocarcinoma).

### 2.2 Total RNA Isolation and Reverse Transcription

Total RNA was isolated from each cell line with the use of TRIzol™ reagent (Ambion™, Thermo Fisher Scientific Inc., Waltham, MA, USA), according to the manufacturer’s guidelines. All RNA samples were diluted in THE RNA Storage Solution (Ambion™) and, subsequently, their concentration and purity were assessed spectrophotometrically at 260 and 280 nm, using a BioSpec-nano micro-volume UV-vis spectrophotometer (Shimadzu, Kyoto, Japan). Then, positive selection of the mRNAs was carried out with NEBNext^®^ Poly(A) mRNA Magnetic Isolation Module (New England Biolabs, Inc) from 5 μg of each total RNA sample.

In the next step, first-strand cDNA synthesis was performed for every mRNA-enriched sample. For this purpose, an oligo-dT_20_ was used as a primer to anneal in the 3' poly(A) tail of the mRNA molecules. Briefly, the cDNA synthesis was performed in reaction volumes of 20 μl, using 2 μg of total RNA, 1 μl of oligo-dT_20_ (10 μM), 1 μl dNTP mix (10 mM each), 4 μl 5X first-strand buffer, 1 μl DTT (0.1 M), 1 μl (40 U) RNaseOUT™ (Invitrogen™, Thermo Fisher Scientific Inc.), and 1 μl (200 U) of SuperScript™ III reverse transcriptase (Invitrogen™, Thermo Fisher Scientific Inc.), following the manufacturers’ protocols. The housekeeping gene glyceraldehyde 3-phosphate dehydrogenase (GAPDH) was used for the quality control of the produced cDNA samples. Finally, equimolar amounts of each cDNA sample were mixed for the creation of 14 distinct cDNA pools that corresponded to a specific human tissue or malignancy. The derived cDNA pools were exploited as templates for the downstream PCR amplification step.

### 2.3 PTEN Amplification with Touchdown PCR

A touchdown PCR-based assay was conducted to amplify the PTEN transcript variants with increased sensitivity, specificity, and PCR yield [37, 38]. For this purpose, two gene-specific primers (GSPs) were designed. The forward GSP (F: 5' – GGAGATATCAAGAGGATGGATTCGAC – 3') was designed to target the first exon of PTEN, whereas the reverse GSP (R: 5' – GTTGAACTGCTAGCCTCTGGATT – 3') was designed to anneal to the last exon.

The touchdown PCR was performed in reaction volumes of 25 μL, containing KAPA Taq buffer A (Kapa Biosystems Inc.), which included MgCl_2_ at a final concentration of 1.5 mM, 0.2 mM dNTPs, 0.4 μM of each primer, and 1 unit of KAPA Taq DNA polymerase (Kapa Biosystems Inc.) in a Veriti 96-well fast thermal cycler (Applied Biosystems™). In addition, the applied cycling protocol that was applied included an initial denaturation step at 95°C for 3 min, followed by 35 cycles of 95°C for 30 s, 65°C (auto-ΔTa: −0.3°C/cycle) for 30 s, 72°C for 2 min, and a final extension step at 72°C for 5 min (Supplementary Fig. **1**). Finally, the produced amplicons were purified using the NucleoSpin^®^ gel and PCR clean-up kit (Macherey-Nagel GmbH & Co. KG, Duren, Germany).

### 2.4 Targeted PTEN-seq with Hybrid Sequencing

The 14 purified products were used as input for the construction of barcoded libraries for nanopore sequencing, which corresponded to amplified PTEN mRNAs from breast cancer, ovarian cancer, prostate cancer, colorectal cancer, urinary bladder cancer, hepatocellular carcinoma, lung cancer, cervical cancer, brain cancer, hematological malignancies, gastric cancer, endometrial cancer, renal cancer, and non-cancerous cell lines. Briefly, NEBNext^®^ Ultra™ II End Repair/dA-Tailing Module (New England Biolabs, Inc.) was used for the end-repair, whereas the adaptor ligation was performed with Quick T4 Ligase (New England Biolabs, Inc.). The barcoded libraries were quantified using the Qubit^®^ 2.0 fluorometer and were mixed equimolarly for the creation of the final mixed library. The MinION Mk1C sequencer (Oxford Nanopore Technologies Ltd., Oxford, UK) was used for the nanopore sequencing run, along with a FLO-MIN106D flow cell (R9.4.1 chemistry) and the ligation sequencing kit (SQK-LSK109, ONT), following the protocol of the manufacturer.

Additionally, the same starting material was used for the generation of an NGS library, which was constructed with NEBNext^®^ Fast DNA Fragmentation and Library Prep Set for Ion Torrent™ (New England Biolabs, Inc.). The preparation and enrichment of the library template were carried out with the Ion PGM™ Hi-Q™ View OT2 kit (Ion Torrent™), whereas the NGS run was conducted on an Ion Personal Genome Machine™ (PGM™) platform with the Ion PGM™ Hi-Q™ View sequencing kit.

### 2.5 Read Polishing and Bioinformatics Analysis

The demultiplexing and basecalling of the raw nanopore sequencing data were performed with Guppy [39]. Only the sequencing reads that exceeded the minimal quality score were further analyzed for the investigation of PTEN splicing. Next, a long-read polishing step was implemented with hybrid error correction algorithms that utilized both the raw nanopore sequencing data and the short-read NGS dataset. The corrected long-reads from each barcoded nanopore sequencing library were aligned to the human reference genome (GRCh38) with Minimap2 [40], whereas the Integrative Genomics Viewer (IGV) was used for the visualization of the successfully aligned reads [41]. The transcriptional profile of PTEN was investigated using specialized algorithms for splice variant detection and quantification [42, 43], as well as in-house developed algorithms [44].

### 2.6 *In Silico* ORF and Gene Ontology Prediction

The predicted open reading frames (ORFs) of the identified PTEN mRNA transcripts were annotated using the online translation platform of ExPASy [45]. Each putative ORF was investigated for the presence of conserved motifs, whereas LncADeep was used to estimate the probability of each identified PTEN transcript to represent long non-coding RNA (lncRNA) [46]. Finally, Gene Ontology (GO) terms with significant prediction scores were provided by DeepGOWeb [47].

## 3 RESULTS

### 3.1 Nanopore Sequencing Unveiled the Transcriptional Profile of PTEN

**Fig. (1):**
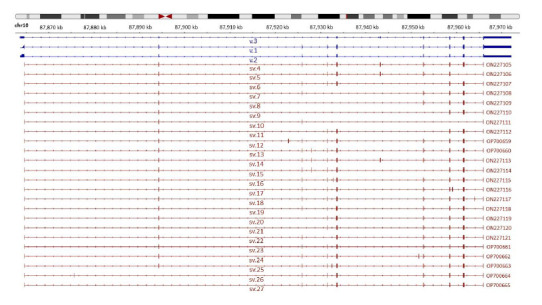
Visualization of the aligned sequencing reads representing novel transcript variants of the human PTEN gene *via* Integrative Genomics Viewer (IGV) tool.

Besides validating the annotated PTEN transcript variants, visualization of the aligned nanopore sequencing reads confirmed the existence of novel splice junctions between annotated exons as well as a total of eight novel cryptic exons. These findings were also confirmed by the utilized isoform detection tools and our in-house developed algorithm [44]. It should be mentioned that the described findings have been derived from 38 human cancerous (SH-SY5Y, H4, U-87 MG, U-251 MG, SK-BR-3, BT-20, MCF-7, BT-474, HeLa, SiHa, DLD-1, HT-29, Caco-2, HCT 116, SW620, 786-O, Caki-1, Hep G2, HuH-7, A549, Raji, SU-DHL-1, ES-2, SK-OV-3, OVCAR-3, MDAH-2774, PC-3, DU 145, LNCaP, HL-60, AGS, RT4, T24, Ishikawa and SK-UT-1B) as well as 4 non-cancerous (HEK-293, 1.2B4, HaCaT and BJ) cell lines. Overall, the present study has identified twenty-four novel PTEN splice variants (PTEN sv.4 – sv.27) whose nucleotide sequences were deposited in GenBank^®^ (Fig. **1**). Due to the long-read technology that nanopore sequencing offers, the derived sequencing reads that encompass the novel splice junctions covered the entire cDNA sequences from the first until the last exon of each PTEN mRNA, thus representing novel PTEN mRNA transcripts (Table **1**).

### 3.2 New Exon-skipping Events Generated a Spectrum of Novel PTEN Splice Variants

**Fig. (2):**
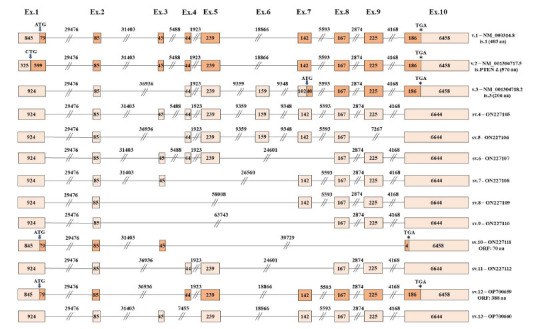
Detailed structure of the newly identified PTEN transcript variants comprising the annotated exons. Exons are shown as rectangles and introns as lines. The length of every exon/intron is also shown. The highlighted regions in rectangles depict the ORFs in each transcript variant. Finally, the positions of the annotated initiation and stop codons are indicated with arrows (↓) and asterisks (*) accordingly.

Based on the obtained raw nanopore sequencing reads, a variety of cassette exon splicing events led to the generation of multiple previously uncharacterized PTEN mRNAs (PTEN sv.4–sv.13). Interestingly, the identified PTEN sv.4 encompassed all the annotated exons of PTEN gene, thus having an increased length as compared to the main transcript, PTEN v.1 (Fig. **2**). Following, PTEN sv.5 completely lacked two annotated exons (exons 3 and 9), containing the novel splicing event between exons 8 and 10. Furthermore, our findings have indicated the existence of two additional splice variants, PTEN sv.6 and sv.11. These mRNAs shared the new splicing event between exons 5 and 8; however, they were differentiated since PTEN v.11 also lacked exon 3.

Two additional novel splice variants, PTEN sv.7–sv.8, were characterized by previously undescribed splicing events that involve exon 7. In specific, exon 7 was found to be alternatively spliced with exon 3 in PTEN sv.7 and with exon 2 in PTEN sv.8 (Fig. **2**). Similarly, exon skipping events between even more distant exons led to the generation of PTEN sv.9–sv.10. Briefly, PTEN sv.9 included the previously unidentified splicing event between exons 2 and 8, whereas PTEN sv.10 was characterized by the novel splice junction between exons 3 and 10. Finally, the identified PTEN sv.12 demonstrated high structural similarity with the annotated PTEN v.3; however, it also lacked exon 6, whereas PTEN sv.13 exhibited a similar nucleotide sequence with PTEN v.1, but additionally, it lacked exon 4.

The *in silico* ORF query analysis on the nucleotide sequences of the identified PTEN mRNAs strongly suggested that besides PTEN sv.10 and sv.12, all the aforementioned splice variants did not utilize the annotate initiation codon that resided on exon 1, since they contained premature termination codons (PTCs). On the contrary, PTEN sv.10 was predicted to contain a small ORF (smORF) of 70 aa and therefore encode a rather non-functional peptide, while PTEN sv.12 harbored an ORF of 388 aa and most likely encoded a novel isoform.

### 3.3 Identification of PTEN mRNA Transcripts with Cryptic Exons

**Fig. (3):**
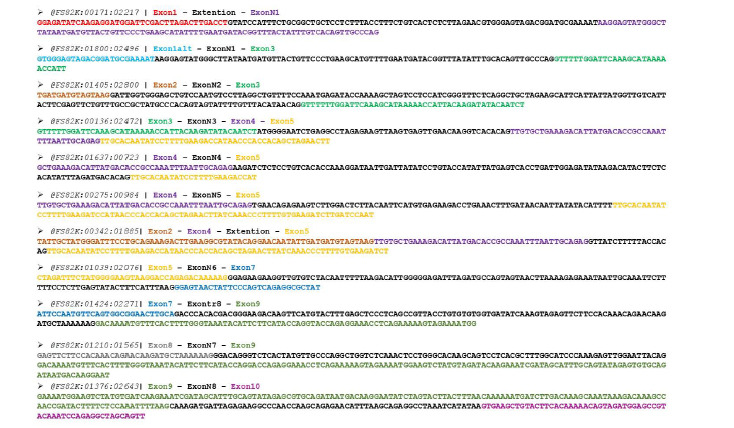
Next-generation sequencing reads that support the existence of novel PTEN exons and alternative splice sites. For visual purposes, the nucleotide bases of each exon are demonstrated in a different color.

**Fig. (4):**
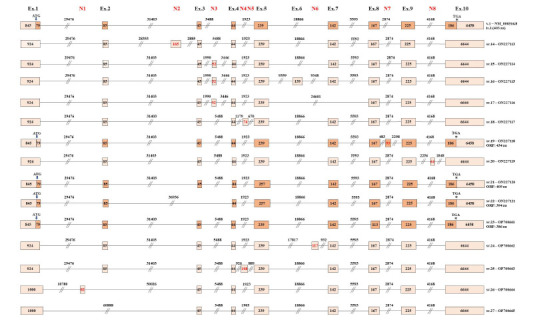
Detailed structure of the novel PTEN transcripts that include newly detected cryptic exons. Exons are shown as rectangles and introns as lines. The length of every exon/intron is also shown. The highlighted regions in rectangles depict the ORFs in each transcript variant. Finally, the positions of the annotated initiation and stop codons are indicated with arrows (↓) and asterisks (*) accordingly.

Besides the described exon skipping events, bioinformatics analysis revealed eight novel exons (named N1–N8), which were successfully aligned by both short- and long-read datasets (Fig. **3**). The existence of N1–N8 led to the identification of fourteen previously uncharacterized mRNAs. Based on the nanopore sequencing data, N1 was located between the annotated exons 1 and 2, being 82 nt in length, while N2 resided between exons 2 and 3, having a total length of 165 nt (Fig. **4**). Our findings support that N1 and N2 were included in the nucleotide sequences of PTEN sv.26 and sv.14, respectively. PTEN sv.14 was identical to the annotated PTEN v.1 downstream of exon 3, but it was differentiated since it contained the sequence of N2.

Additionally, N3 was located between exons 3 and 4, being 52 nt in length and existing in three previously unidentified transcripts, PTEN sv.15–sv.17 (Fig. **4**). Briefly, sv.15 and sv.16 shared the same nucleotide sequence with the annotated transcript PTEN v.1 and sv.4, accordingly, but also included N3, while sv.17 was characterized by the previously undescribed splicing event between exons 5 and 8. Furthermore, the acquired datasets revealed the existence of five novel transcripts, PTEN sv.18–sv.20 and sv.24–sv.25, each one of which possessed one additional novel cryptic exon (N4–N8).

### 3.4 New PTEN mRNAs Mediated by Alternative 5' and 3' Splice Sites

Even though exon skipping was found to be the predominant mechanism leading to a wide spectrum of previously undescribed PTEN mRNAs, our findings have supported the involvement of alternative 5' and 3' splice sites in the generation of novel splice variants. In the case of the identified PTEN sv.23, an alternative 3' splice site on exon 8 was utilized, leading to a truncated exon 8, being 113 nt in length. Upstream and downstream of exon 8, sv.23 was identical to the annotated PTEN v.1 (Fig. **4**). Similarly, the newly identified PTEN sv.21 and sv.22 were derived from an alternative 5' splice site of exon 5, which resulted in an extended exon 5 that was 257 nt in size instead of 239 nt. Finally, PTEN sv.26 and sv.27 shared an alternative 5' splice site of exon 1, and their subtle differentiation corresponded to the existence of N1, which was absent from sv.27 (Fig. **4**).

### 3.5 Transcriptional Landscape of PTEN mRNAs in Multiple Human Malignancies

**Fig. (5):**
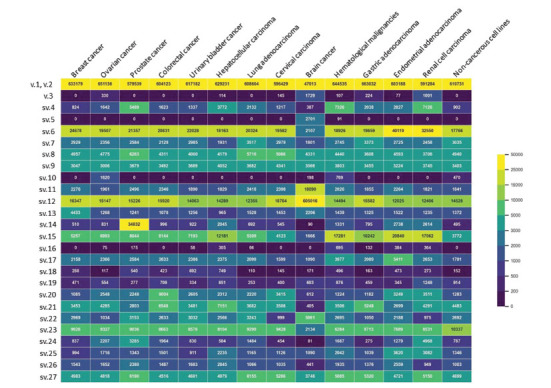
Heatmap indicating the relative abundance of each PTEN transcript compared to the main mRNA. Yellow coloring is indicative of a higher relative abundance of the corresponding transcript variant, whereas purple coloring corresponds to low-frequent or even undetectable transcripts.

Demultiplexing of the nanopore sequencing data and expression analysis revealed that the described PTEN splice variants demonstrated broad expression patterns across human tissues (Fig. **5**). As expected, the previously uncharacterized splice variants appeared in notably lower frequency as compared to the main PTEN v.1. Interestingly, based on the read-per-million (RPM) values, most of the novel transcripts were characterized by higher abundance as compared to the annotated PTEN v.3, a fact that enhances the significance of the presented findings. In detail, sv.6, sv.12, and sv.15 were identified as the most abundant among the human cell lines that were tested. Additionally, it is worth mentioning that some of the new transcripts demonstrated a significant, tissue-specific expression. More specifically, sv.11 and sv.12 were highly expressed in brain cancer, and surprisingly, sv.12 was found to be more abundant than the main transcript PTEN v.1 (Fig. **5**). On the other hand, sv.5 seemed to be expressed in low levels only in the cell lines corresponding to brain cancer, since it was undetectable in the rest of them, based on the sequencing depth. Of note, sv.6 was one of the most abundant transcripts in almost all tissues that were tested, but it demonstrated low expression levels in brain cancer. Finally, the novel transcript sv.14 was overexpressed in prostate cancer, while demonstrating low expression levels in the rest of the human tissues examined.

### 3.6 *In Silico* Identification of Novel Open Reading Frames

Although the identification of new ORFs in the present study was limited to computational analysis, it was worthwhile to investigate whether some of the described new mRNA transcripts harbored ORFs capable of translating novel PTEN isoforms with defined functional roles. The assessment of the translational capability of each splice variant was based on the rule of the nonsense-mediated mRNA decay (NMD) pathway, which supports that mRNAs with termination codons residing in the last exon or 50 - 55 nucleotides upstream of the last splice junction most likely escaped the NMD and translated into proteins [48]. Initially, an ORF query was performed evaluating the existence of putative ORFs oriented by the annotated initiation codon that characterized the main PTEN v.1 (Supplementary Fig. **2**). The analysis showed PTEN sv.12, sv.19, sv.21, sv.22, and sv.23 to be highly promising to encode novel protein isoforms demonstrating catalytic activity, since their putative amino acid sequence was highly similar to the main PTEN isoform (Figs. **2** and **4**). However, the potential translation of these transcripts by non-canonical ORFs remains a possibility and merits further investigation [14]. For instance, sv.6 and sv.11 were predicted to encode the same protein isoform, a novel protein of 221 aa, using an alternative initiation codon. Likewise, the potential translation of transcripts sv.14 and sv.15 generated a new protein isoform of 270 aa. The subsequent alignment of these novel isoforms’ amino acid sequence against the known PTEN proteins revealed that these molecules resembled the annotated isoform 3 in terms of their primary structure.

### 3.7 Functional Annotation of the Novel Protein-coding PTEN mRNAs

**Fig. (6):**
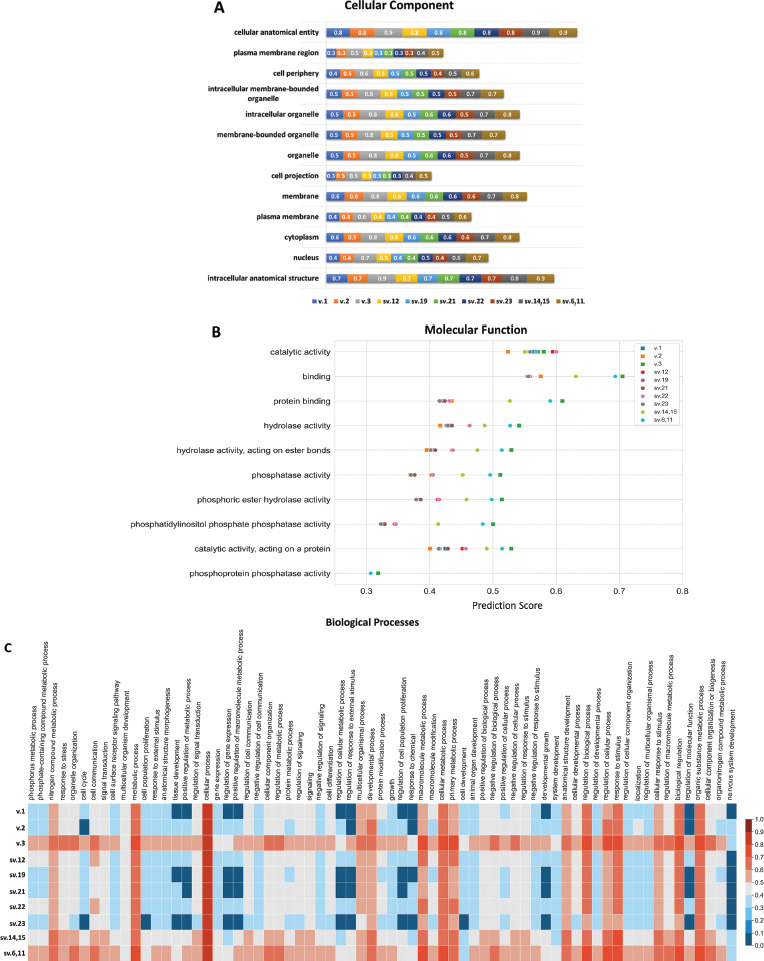
Gene ontology analysis of the putative new isoforms encoded by the described PTEN mRNA transcripts. (**A**) Plot demonstrating the GO scores of each predicted new isoform in terms of cellular components. (**B**) Scatterplot providing the GO scores for the molecular function. (**C**) Heatmap showing the probability of each putative PTEN isoform to participate in a wide spectrum of biological processes.

To evaluate the functionality of the predicted PTEN proteins, we performed Gene Ontology (GO) analysis. More specifically, the obtained GO results indicated that in terms of cellular localization, the encoded isoforms from sv.19, sv.21, and sv.22 demonstrated an identical pattern to the main PTEN isoform (403 aa) as well as PTEN-L (Fig. **6A**). Similarly, the putative PTEN isoform encoded by sv.6 and sv.11 was expected to be detected in almost all compartments where PTEN isoform 3 is localized (Fig. **6A**). Regarding the molecular function, the putative isoforms encoded by sv.19, sv.21, and sv.23 were predicted to have the same functionality as the main PTEN isoform and PTEN-L (Fig. **6B**). Based on the obtained GO prediction scores, the protein isoform corresponding to sv.6 and sv.11 demonstrated a similar pattern to the PTEN isoform 3, suggesting that these isoforms are highly expected to function in the same manner. Interestingly, all the putative proteins were predicted to exhibit catalytic activity, with scores ranging from 0.5-0.6, which also characterized the main isoform (Fig. **6B**). Finally, all putative proteins were characterized by high GO scores in terms of cellular and metabolic processes, primary metabolic process, regulation of biological processes, response to stimulus, biological regulation, and organic substance metabolic process, which indicated that these putative molecules are highly promising to exhibit identical biological processes (Fig. **6C**).

## 4 DISCUSSION

PTEN/PI3K/AKT constitutes a significant signaling pathway that regulates numerous biological processes, such as cell growth and proliferation, metabolism, and apoptosis, contributing to the maintenance of the physiological state of cells [12, 22, 23, 49, 50]. PTEN is a dual phosphatase with both protein and lipid phosphatase activity, which uses mainly PIP3 as its substrate, sustaining its low levels in the membrane [22, 40, 51, 52]. Since most of the molecules involved in the described biological pathway have been described as key regulators in cancer, a multilevel study of their genes is crucial. Although many previous works have investigated the physiological role of PTEN protein in normal conditions, as well as its involvement in cancer development and progression, the transcriptional profile of PTEN has been poorly investigated [5, 9, 27]. By deciphering the mutational profile of PTEN in various cancers and evaluating its impact on the translated isoforms, the study of this gene at the transcriptional level is imperative [1, 5, 10, 28-30, 53-55].

In the present study, the design and implementation of a barcoded targeted nanopore sequencing approach with enhanced sequencing depth and polished long reads allowed the detection of novel PTEN transcripts in multiple cancer types as well as non-cancerous cells and the evaluation of their expression levels. Of note, rather than conducting the “gold-standard” direct RNA sequencing method that fails to detect less abundant novel mRNAs due to the decreased sequencing depth, a PCR amplification step was performed prior to nanopore sequencing to render PTEN transcripts with lower representation detectable. The obtained results led to the identification of 24 novel PTEN transcript variants, 10 of which were predicted to have ORFs and thus encode new protein isoforms utilizing the annotated or alternative initiation and stop codons.

The identification of the described novel PTEN splice variants has several significant implications for cancer biology. Firstly, these variants may contribute to PTEN dysregulation in cancer. PTEN mutations and deletions are well-established drivers of tumorigenesis, but they only account for a fraction of PTEN alterations in cancer. The expression of novel PTEN splice variants may be a more prevalent mechanism of PTEN dysregulation in cancer, which may have implications for diagnosis and treatment. Secondly, these variants may have distinct functions and activities from the main PTEN protein, which may affect their tumor suppressor or oncogenic properties [18, 21, 56]. PTEN-L has been shown to regulate mitochondrial function, which is critical for cellular energy metabolism and survival [14, 57, 58]. PTENβ has been shown to act as a transcriptional regulator of genes involved in cell survival and proliferation [16, 21]. These activities are distinct from the lipid phosphatase activity of the main PTEN protein, suggesting that these novel splice variants may have additional functions that are not shared by the main PTEN protein. Thirdly, the detection of these splice variants raises questions regarding the regulation of PTEN mRNA splicing and the mechanisms that govern the generation of these variants. The 5' UTR of PTEN-L contains a sequence that is conserved across species and is predicted to be involved in the regulation of PTEN mRNA translation. The identification of this regulatory sequence raises the possibility that other regulatory sequences may exist in the PTEN gene.

Since the newly identified PTEN transcripts are predicted to encode new protein isoforms, the repertoire of PTEN functions is widening. The existence of PTEN isoforms that exhibit novel properties and carry out additional functions will undoubtedly shed light on the application of this tumor suppressor gene in tumor development and cell dysregulation. Interestingly, the results obtained from Gene Ontology (GO) functional analysis suggested the putative proteins corresponding to the novel PTEN transcript variants sv.12, sv.19, sv.21, and sv.22, to be detected in all cellular compartments where PTEN and *L*-PTEN are localized, enhancing their potential function in these compartments. Likewise, the putative PTEN protein isoform encoded by transcript variants sv.6 and sv.11 was predicted to be localized in almost all cellular compartments where PTEN isoform 3 was detected, indicating that PTEN protein isoforms could be sub-grouped regarding their functionality which, according to previous studies, is not limited in the phosphorylation of lipids. It is noteworthy that all putative proteins are predicted to have catalytic and phosphatase activity, highlighting their potential function as phosphatases. Regarding the biological processes in which these molecules are involved, all putative proteins have exhibited high GO scores in cellular and metabolic processes, primary metabolic processes, regulation of biological processes, response to stimuli, biological regulation, and metabolic processes of organic substances, indicating that these putative molecules are highly likely to be involved in the same biological processes in which the annotated PTEN proteins are involved (Fig. **6C**). However, the obtained GO results suggested the involvement of the putative PTEN proteins in different pathways, confirming the multifaced role of PTEN in physiological and pathological conditions (Figs. **6B**, **6C**). In addition to the new insights gained from the GO functional analysis, the significance of the present study lies in the decoding of PTEN translational landscape.

Interestingly, the newly identified PTEN transcript variants showed broad expression patterns in the human tissues studied, both in cancerous and non-cancerous cell lines, clearly indicating that these results cannot be artifacts. As expected, most of the identified PTEN transcripts exhibited lower expression levels compared to the main transcript PTEN v.1, whereas their abundance was higher than the annotated transcript variant PTEN v.3 (Fig. **5**), confirming the poorly investigated transcriptional profile of this gene. Interestingly, most of the new PTEN variants were also detectable in non-cancerous cell lines, supporting their existence and ruling out the possibility that they are merely combinations of splicing errors due to dysregulation of the splicing machinery in cancer. On the other hand, sv.5 and sv.10 have counts in only a few tissues and represent ambiguous transcripts that need further investigation. PTEN transcripts sv.6 and sv.12 seem to be the most highly expressed mRNAs among the novel PTEN transcript variants that were identified. Of note, sv.12 has been found to be overexpressed in brain cancer and, astonishingly, it displayed higher expression levels than the main variant in this cancer type. Furthermore, sv.11 and sv.14 were found to be over expressed in brain and prostate cancer, respectively, strengthening their possible involvement in these cancers. However, the potential expression of these transcript variants in other tumors that were not included in the present study cannot be ruled out. Nevertheless, considering that the panel of the cell lines used was broad, we could gain an accurate background regarding the diverse PTEN expression patterns among them. The tumor-specific expression pattern of this gene indicates that these “tumor-specific” transcripts can be used as potential biomarkers for diagnosis and/or staging of a specific cancer, or represent potential therapeutic targets. When questioning whether these transcripts are related to PTEN transcriptional regulation or certain tumor-specific RNA splicing-related signals, we assume that the most feasible scenario is the second one. Since there are “signatures” in the mRNA molecules that spliceosomes recognize and act on, gene mutations can modify these signatures, or dysregulation of spliceosomes can lead to disregarding these signatures, affecting the splicing mechanism. So, the generation of tumor-specific transcript variants can be an effect of tumor-specific gene mutations or spliceosome dysregulation.

Since the previous studies conducted have focused on the mutational analysis of PTEN, little is known regarding the transcriptional profile of this gene. To date, two PTEN mRNA transcripts have been identified and their translation generates four different protein isoforms, the main PTEN protein, PTEN-L [15], PTENβ [16] and PTEN is. 3. Since the data on the transcriptional landscape of PTEN are limited, our study has revealed for the first time aspects of the PTEN gene that have not been explored before, shedding some light on the dysregulation of this tumor-suppressor gene in cancer.

## CONCLUSION

In conclusion, our study has highlighted the wide spectrum of previously unknown PTEN mRNA transcripts and evaluated their expression pattern in a broad panel of cancerous and non-cancerous human cell lines, elucidating the involvement of PTEN in the dysregulation of the cell cycle and thus in tumor development. The identification of novel PTEN splice variants with ORFs and similar functions to the main mRNA has significant implications for understanding PTEN regulation, function, and cancer biology. These variants may contribute to PTEN dysregulation in cancer, have distinct functions and activities from the main PTEN protein, and raise questions about the regulation of PTEN mRNA splicing. Further exploration of the splice variants described could provide new insights into PTEN biology and open new avenues for the diagnosis and treatment of PTEN-related diseases, including cancer.

## Figures and Tables

**Table 1:** Summary of the newly identified *PTEN* transcripts.

Exon Transcript	**1**	**alt1**	**N1**	**2**	**N2**	**3**	**N3**	**4**	**N4**	**N5**	**alt5**	**5**	**6**	**N6**	**7**	**8**	**alt8**	**N7**	**9**	**N8**	**10**
**sv.4**	✓	-	-	✓	-	✓	-	✓	-	-	-	✓	✓	-	✓	✓	-	-	✓	-	✓
**sv.5**	✓	-	-	✓	-	-	-	✓	-	-	-	✓	✓	-	✓	✓	-	-	-	-	✓
**sv.6**	✓	-	-	✓	-	✓	-	✓	-	-	-	✓	-	-	-	✓	-	-	✓	-	✓
**sv.7**	✓	-	-	✓	-	✓	-	-	-	-	-	-	-	-	✓	✓	-	-	✓	-	✓
**sv.8**	✓	-	-	✓	-	-	-	-	-	-	-	-	-	-	✓	✓	-	-	✓	-	✓
**sv.9**	✓	-	-	✓	-	-	-	-	-	-	-	-	-	-	-	✓	-	-	✓	-	✓
**sv.10**	✓	-	-	✓	-	✓	-	-	-	-	-	-	-	-	-	-	-	-	-	-	✓
**sv.11**	✓	-	-	✓	-	-	-	✓	-	-	-	✓	-	-	-	✓	-	-	✓	-	✓
**sv.12**	✓	-	-	✓	-	-	-	✓	-	-	-	✓	-	-	✓	✓	-	-	✓	-	✓
**sv.13**	✓	-	-	✓	-	✓	-	-	-	-	-	✓	-	-	✓	✓	-	-	✓	-	✓
**sv.14**	✓	-	-	✓	✓	✓	-	✓	-	-	-	✓	-	-	✓	✓	-	-	✓	-	✓
**sv.15**	✓	-	-	✓	-	✓	✓	✓	-	-	-	✓	-	-	✓	✓	-	-	✓	-	✓
**sv.16**	✓	-	-	✓	-	✓	✓	✓	-	-	-	✓	✓	-	✓	✓	-	-	✓	-	✓
**sv.17**	✓	-	-	✓	-	✓	✓	✓	-	-	-	✓	-	-	-	✓	-	-	✓	-	✓
**sv.18**	✓	-	-	✓	-	✓	-	✓	-	✓	-	✓	-	-	✓	✓	-	-	✓	-	✓
**sv.19**	✓	-	-	✓	-	✓	-	✓	-	-	-	✓	-	-	✓	✓	-	✓	✓	-	✓
**sv.20**	✓	-	-	✓	-	✓	-	✓	-	-	-	✓	-	-	✓	✓	-	-	✓	✓	✓
**sv.21**	✓	-	-	✓	-	✓	-	✓	-	-	✓	-	-	-	✓	✓	-	-	✓	-	✓
**sv.22**	✓	-	-	✓	-	-	-	✓	-	-	✓	-	-	-	✓	✓	-	-	✓	-	✓
**sv.23**	✓	-	-	✓	-	✓	-	✓	-	-	-	✓	-	-	✓	-	✓	-	✓	-	✓
**sv.24**	✓	-	-	✓	-	✓	-	✓	-	-	-	✓	-	✓	✓	✓	-	-	✓	-	✓
**sv.25**	✓	-	-	✓	-	✓	-	✓	✓	-	-	✓	-	-	✓	✓	-	-	✓	-	✓
**sv.26**	-	✓	✓	-	-	✓	-	✓	-	-	-	✓	-	-	✓	✓	-	-	✓	-	✓
**sv.27**	-	✓	-	-	-	✓	-	✓	-	-	-	✓	-	-	✓	✓	-	-	✓	-	✓

## Data Availability

The datasets of the present study are available from the corresponding author upon reasonable request.

## LIST OF ABBREVIATIONS

ATCC: American Type Culture Collection
GAPDH: Glyceraldehyde 3-phosphate Dehydrogenase
GO: Gene Ontology
GSPs: Gene-specific Primers
H3K4: H3 lysine 4
IGV: Integrative Genomics Viewer
lncRNA: Long Non-coding RNA
NGS: Next-generation Sequencing
NMD: Nonsense-mediated mRNA Decay
ONT: Oxford Nanopore Technologies
ORFs: Open Reading Frames
PBD: PIP2-binding Domain
PDK: 3phosphoinositide-dependent Kinase
PTCs: Premature Termination Codons
PTEN: Phosphatase and Tensin Homolog
PTEN-L: PTEN-Long
PTPs: Protein Tyrosine Phosphatases
RPM: Read-per-million
smORF: small ORF
TGS: Third-generation Sequencing

## References

[1] Bigner S.H., Mark J., Mahaley M.S., Bigner D.D. (1984). Patterns of the early, gross chromosomal changes in malignant human gliomas.. Hereditas.

[2] Li J., Yen C., Liaw D., Podsypanina K., Bose S., Wang S.I., Puc J., Miliaresis C., Rodgers L., McCombie R., Bigner S.H., Giovanella B.C., Ittmann M., Tycko B., Hibshoosh H., Wigler M.H., Parsons R. (1997). PTEN, a putative protein tyrosine phosphatase gene mutated in human brain, breast, and prostate cancer.. Science.

[3] Steck P.A., Pershouse M.A., Jasser S.A., Yung W.K.A., Lin H., Ligon A.H., Langford L.A., Baumgard M.L., Hattier T., Davis T., Frye C., Hu R., Swedlund B., Teng D.H.R., Tavtigian S.V. (1997). Identification of a candidate tumour suppressor gene, MMAC1, at chromosome 10q23.3 that is mutated in multiple advanced cancers.. Nat. Genet..

[4] Podsypanina K., Ellenson L.H., Nemes A., Gu J., Tamura M., Yamada K.M., Cordon-Cardo C., Catoretti G., Fisher P.E., Parsons R. (1999). Mutation of Pten/Mmac1 in mice causes neoplasia in multiple organ systems.. Proc. Natl. Acad. Sci. USA.

[5] Suzuki A., de la Pompa J.L., Stambolic V., Elia A.J., Sasaki T., Barrantes I.B., Ho A., Wakeham A. (1998). ltie, A.; Khoo, W.; Fukumoto, M.; Mak, T.W. High cancer susceptibility and embryonic lethality associated with mutation of the PTEN tumor suppressor gene in mice.. Curr. Biol..

[6] Cristofano A.D., Pesce B., Cordon-Cardo C., Pandolfi P.P. (1998). Pten is essential for embryonic development and tumour suppression.. Nat. Genet..

[7] Alimonti A., Carracedo A., Clohessy J.G., Trotman L.C., Nardella C., Egia A., Salmena L., Sampieri K., Haveman W.J., Brogi E., Richardson A.L., Zhang J., Pandolfi P.P. (2010). Subtle variations in Pten dose determine cancer susceptibility.. Nat. Genet..

[8] Di Cristofano A., De Acetis M., Koff A., Cordon-Cardo C., Pandolfi P. (2001). P. Pten and p27KIP1 cooperate in prostate cancer tumor suppression in the mouse.. Nat. Genet..

[9] Palomero T., Sulis M.L., Cortina M., Real P.J., Barnes K., Ciofani M., Caparros E., Buteau J., Brown K., Perkins S.L., Bhagat G., Agarwal A.M., Basso G., Castillo M., Nagase S., Cordon-Cardo C., Parsons R., Zúñiga-Pflücker J.C., Dominguez M., Ferrando A.A. (2007). Mutational loss of PTEN induces resistance to NOTCH1 inhibition in T-cell leukemia.. Nat. Med..

[10] Papa A., Wan L., Bonora M., Salmena L., Song M.S., Hobbs R.M., Lunardi A., Webster K., Ng C., Newton R.H., Knoblauch N., Guarnerio J., Ito K., Turka L.A., Beck A.H., Pinton P., Bronson R.T., Wei W., Pandolfi P.P. (2014). Cancer-associated PTEN mutants act in a dominant-negative manner to suppress PTEN protein function.. Cell.

[11] Worby C.A., Dixon J.E. (2014). PTEN.. Annu. Rev. Biochem..

[12] Lee Y.R., Chen M., Pandolfi P.P. (2018). The functions and regulation of the PTEN tumour suppressor: New modes and prospects.. Nat. Rev. Mol. Cell Biol..

[13] Heinrich F., Chakravarthy S., Nanda H., Papa A., Pandolfi P.P., Ross A.H., Harishchandra R.K., Gericke A., Lösche M. (2015). The PTEN Tumor Suppressor Forms Homodimers in Solution.. Structure.

[14] Liang H., He S., Yang J., Jia X., Wang P., Chen X., Zhang Z., Zou X., McNutt M.A., Shen W.H., Yin Y. (2014). PTENα, a PTEN isoform translated through alternative initiation, regulates mitochondrial function and energy metabolism.. Cell Metab..

[15] Hopkins B.D., Fine B., Steinbach N., Dendy M., Rapp Z., Shaw J., Pappas K., Yu J.S., Hodakoski C., Mense S., Klein J., Pegno S., Sulis M.L., Goldstein H., Amendolara B., Lei L., Maurer M., Bruce J., Canoll P., Hibshoosh H., Parsons R. (2013). A secreted PTEN phosphatase that enters cells to alter signaling and survival.. Science.

[16] Liang H., Chen X., Yin Q., Ruan D., Zhao X., Zhang C., McNutt M.A., Yin Y. (2017). PTENβ is an alternatively translated isoform of PTEN that regulates rDNA transcription.. Nat. Commun..

[17] Liang H., Yin Y. (2019). Multiple roles of PTEN isoforms PTENα and PTENβ in cellular activities and tumor development.. Sci. China Life Sci..

[18] Zhang C., Ma H.M., Dong S.S., Zhang N., He P., Ge M.K., Xia L., Yu J.X., Xia Q., Chen G.Q., Shen S.M. (2022). Furin extracellularly cleaves secreted PTENα/β to generate C-terminal fragment with a tumor-suppressive role.. Cell Death Dis..

[19] Taylor J., Abdel-Wahab O. (2019). PTEN isoforms with dual and opposing function.. Nat. Cell Biol..

[20] Eldeeb M.A., Esmaili M., Hassan M., Ragheb M.A. (2022). The role of PTEN-L in modulating PINK1-Parkin-mediated mitophagy.. Neurotox. Res..

[21] Shen S.M., Zhang C., Ge M.K., Dong S.S., Xia L., He P., Zhang N., Ji Y., Yang S., Yu Y., Zheng J.K., Yu J.X., Xia Q., Chen G.Q. (2019). PTENα and PTENβ promote carcinogenesis through WDR5 and H3K4 trimethylation.. Nat. Cell Biol..

[22] Fruman D.A., Rommel C. (2014). PI3K and cancer: Lessons, challenges and opportunities.. Nat. Rev. Drug Discov..

[23] Manning B.D., Cantley L.C. (2007). AKT/PKB signaling: Navigating downstream.. Cell.

[24] Pulido R. (2015). PTEN: A yin-yang master regulator protein in health and disease.. Methods.

[25] Perevalova A.M., Kobelev V.S., Sisakyan V.G., Gulyaeva L.F., Pustylnyak V.O. (2022). Role of tumor suppressor PTEN and its regulation in malignant transformation of endometrium.. Biochemistry.

[26] Shimobayashi M., Hall M.N. (2014). Making new contacts: the mTOR network in metabolism and signalling crosstalk.. Nat. Rev. Mol. Cell Biol..

[27] Leslie N.R., Downes C.P. (2004). PTEN function: How normal cells control it and tumour cells lose it.. Biochem. J..

[28] Bonneau D., Longy M. (2000). Mutations of the human PTEN gene.. Hum. Mutat..

[29] Mighell T.L., Evans-Dutson S., O’Roak B.J. (2018). A saturation mutagenesis approach to understanding PTEN lipid phosphatase activity and genotype-phenotype relationships.. Am. J. Hum. Genet..

[30] Rodríguez-Escudero I., Oliver M.D., Andrés-Pons A., Molina M., Cid V.J., Pulido R. (2011). A comprehensive functional analysis of PTEN mutations: Implications in tumor- and autism-related syndromes.. Hum. Mol. Genet..

[31] Denu J.M., Stuckey J.A., Saper M.A., Dixon J.E. (1996). Form and function in protein dephosphorylation.. Cell.

[32] Lee J.O., Yang H., Georgescu M.M., Di Cristofano A., Maehama T., Shi Y., Dixon J.E., Pandolfi P., Pavletich N.P. (1999). Crystal structure of the PTEN tumor suppressor: Implications for its phosphoinositide phosphatase activity and membrane association.. Cell.

[33] Adamopoulos P.G., Kontos C.K., Scorilas A. (2017). Molecular cloning of novel transcripts of human kallikrein-related peptidases 5, 6, 7, 8 and 9 (KLK5 – KLK9), using Next-generation sequencing.. Sci. Rep..

[34] van Dijk E.L., Jaszczyszyn Y., Naquin D., Thermes C. (2018). The third revolution in sequencing technology.. Trends Genet..

[35] Athanasopoulou K., Boti M.A., Adamopoulos P.G., Skourou P.C., Scorilas A. (2021). Third-Generation Sequencing: The spearhead towards the radical transformation of modern genomics.. Life.

[36] Michael T.P., Jupe F., Bemm F., Motley S.T., Sandoval J.P., Lanz C., Loudet O., Weigel D., Ecker J.R. (2018). High contiguity Arabidopsis thaliana genome assembly with a single nanopore flow cell.. Nat. Commun..

[37] Korbie D.J., Mattick J.S. (2008). Touchdown PCR for increased specificity and sensitivity in PCR amplification.. Nat. Protoc..

[38] Adamopoulos P.G., Tsiakanikas P., Boti M.A., Scorilas A. (2021). Targeted long-read sequencing decodes the transcriptional atlas of the founding RAS gene family members.. Int. J. Mol. Sci..

[39] Wick R.R., Judd L.M., Holt K.E. (2019). Performance of neural network basecalling tools for Oxford Nanopore sequencing.. Genome Biol..

[40] Li H. (2018). Minimap2: pairwise alignment for nucleotide sequences.. Bioinformatics.

[41] Thorvaldsdóttir H., Robinson J.T., Mesirov J.P. (2013). Integrative Genomics Viewer (IGV): High-performance genomics data visualization and exploration.. Brief. Bioinform..

[42] Holmqvist I., Bäckerholm A., Tian Y., Xie G., Thorell K., Tang K.W. (2021). FLAME: Long-read bioinformatics tool for comprehensive spliceome characterization.. RNA.

[43] de la Fuente L., Arzalluz-Luque Á., Tardáguila M., del Risco H., Martí C., Tarazona S., Salguero P., Scott R., Lerma A., Alastrue-Agudo A., Bonilla P., Newman J.R.B., Kosugi S., McIntyre L.M., Moreno-Manzano V., Conesa A. (2020). tappAS: A comprehensive computational framework for the analysis of the functional impact of differential splicing.. Genome Biol..

[44] Adamopoulos P.G., Theodoropoulou M.C., Scorilas A. (2018). Alternative splicing detection tool—a novel PERL algorithm for sensitive detection of splicing events, based on next-generation sequencing data analysis.. Ann. Transl. Med..

[45] Artimo P. (2012). ExPASy: SIB bioinformatics resource portal., Nucleic Acids Res.

[46] Yang C., Yang L., Zhou M., Xie H., Zhang C., Wang M.D., Zhu H. (2018). LncADeep: An ab initio lncRNA identification and functional annotation tool based on deep learning.. Bioinformatics.

[47] Kulmanov M., Hoehndorf R. (2021). DeepGOPlus: Improved protein function prediction from sequence.. Bioinformatics.

[48] Zhang Z., Xin D., Wang P., Zhou L., Hu L., Kong X., Hurst L.D. (2009). Noisy splicing, more than expression regulation, explains why some exons are subject to nonsense-mediated mRNA decay.. BMC Biol..

[49] Vanhaesebroeck B., Stephens L., Hawkins P. (2012). PI3K signalling: The path to discovery and understanding.. Nat. Rev. Mol. Cell Biol..

[50] Stambolic V., Suzuki A., de la Pompa J.L., Brothers G.M., Mirtsos C., Sasaki T., Ruland J., Penninger J.M., Siderovski D.P., Mak T.W. (1998). Negative regulation of PKB/Akt-dependent cell survival by the tumor suppressor PTEN.. Cell.

[51] Durrant T.N., Hutchinson J.L., Heesom K.J., Anderson K.E., Stephens L.R., Hawkins P.T., Marshall A.J., Moore S.F., Hers I. (2017). In-depth PtdIns(3,4,5)P3 signalosome analysis identifies DAPP1 as a negative regulator of GPVI-driven platelet function.. Blood Adv..

[52] Leslie N.R., Dixon M.J., Schenning M., Gray A., Batty I.H. (2012). Distinct inactivation of PI3K signalling by PTEN and 5-phosphatases.. Adv. Biol. Regul..

[53] Maehama T., Dixon J.E. (1998). The tumor suppressor, PTEN/MMAC1, dephosphorylates the lipid second messenger, phosphatidylinositol 3,4,5-trisphosphate.. J. Biol. Chem..

[54] Knudson A.G. (1971). Mutation and cancer: Statistical study of retinoblastoma.. Proc. Natl. Acad. Sci..

[55] Song M.S., Salmena L., Pandolfi P.P. (2012). The functions and regulation of the PTEN tumour suppressor.. Nat. Rev. Mol. Cell Biol..

[56] Liu A., Zhu Y., Chen W., Merlino G., Yu Y. (2022). PTEN dual lipid- and protein-phosphatase function in tumor progression.. Cancers.

[57] Li Y., He L., Zeng N., Sahu D., Cadenas E., Shearn C., Li W., Stiles B.L. (2013). Phosphatase and tensin homolog deleted on chromosome 10 (PTEN) signaling regulates mitochondrial biogenesis and respiration via estrogen-related receptor α (ERRα).. J. Biol. Chem..

[58] Li G., Yang J., Yang C., Zhu M., Jin Y., McNutt M.A., Yin Y. (2018). PTENα regulates mitophagy and maintains mitochondrial quality control.. Autophagy.

