# Supplementary material for: Unraveling the Concealed Transcriptomic Landscape of PTEN in Human Malignancies
Source: Curr Genomics. 2023 Dec 12;24(4):250–62. doi: 10.2174/0113892029265367231013113304 (PMC10758127; doi:10.2174/0113892029265367231013113304)
Supplement: Supplementary file 1 [file CG-24-250_SD1.pdf]

Supplementary Material

Unraveling the Concealed Transcriptomic Landscape of PTEN in Human Malignancies

Michaela A. Boti[1], Panagiotis G. Adamopoulos[1], Dido Vassilacopoulou[1] and Andreas Scorilas[1,*]

[1]*Department of Biochemistry and Molecular Biology, Faculty of Biology, National and Kapodistrian University of Athens, Athens, Greece*

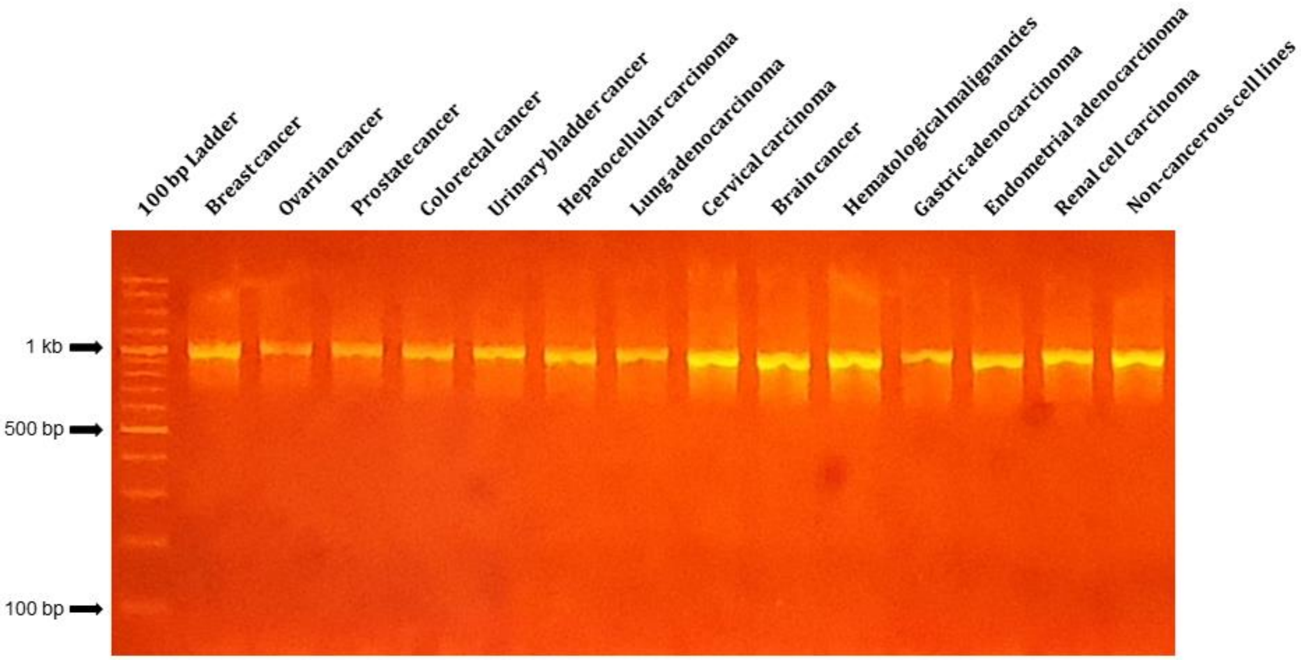

Supplementary Fig. (1). Electrophoresis results demonstrating the generated PTEN-specific amplicon from the implemented touchdown PCR in all the cell lines that were used for the present study.

>PTEN_isoform_1_403aa
MTAIIKEIVSRNKRRYQEDGFDLDLTYIYPNIIAMGFPAERLEGVYRNNIDDVVRFLDSK
HKNHYKIYNLCAERHYDTAKFNCRVAQYPFEDHNPPQLELIKPFCEDLDQWLSEDDNHVA
AIHCKAGKGRTGVMICAYLLHRGKFLKAQEALDFYGEVRTRDKKGVTIPSQRRYVYYYSY
LLKNHLDYRPVALLFHKMMFETIPMFSGGTCNPQFVVCQLKVKIYSSNSGPTRREDKFMY
FEFPQPLPVCGDIKVEFFHKQNKMLKKDKMFHFWVNTFFIPGPEETSEKVENGSLCDQEI
DSICSIERADNDKEYLVLTLTKNDLDKANKDKANRYFSPNFKVKLYFTKTVEEPSNPEAS
SSTSVTPDVSDNEPDHYRYSDTTDSDPENEPFDEDQHTQITKV*

>PTEN_isoform_2_576aa
MERGGEAAAAAAAAAAAPGRGSESPVTISRAGNAGELVSPLLLPPTRRRRRRHIQGPGPV
LNLPSAAAAPPVARAPEAAGGGSRSEDYSSSPHSAAAAARPLAAEEKQAQSLQPSSSRRS
SHYPAAVQSQAAAERGASATAKSRAISILQKKPRHQQLLPSLSSFFFSHRLPDMTAIIKE
IVSRNKRRYQEDGFDLDLTYIYPNIIAMGFPAERLEGVYRNNIDDVVRFLDSKHKNHYKI
YNLCAERHYDTAKFNCRVAQYPFEDHNPPQLELIKPFCEDLDQWLSEDDNHVAAIHCKAG
KGRTGVMICAYLLHRGKFLKAQEALDFYGEVRTRDKKGVTIPSQRRYVYYYSYLLKNHLD
YRPVALLFHKMMFETIPMFSGGTCNPQFVVCQLKVKIYSSNSGPTRREDKFMYFEFPQPL
PVCGDIKVEFFHKQNKMLKKDKMFHFWVNTFFIPGPEETSEKVENGSLCDQEIDSICSIE
RADNDKEYLVLTLTKNDLDKANKDKANRYFSPNFKVKLYFTKTVEEPSNPEASSSTSVTP
DVSDNEPDHYRYSDTTDSDPENEPFDEDQHTQITKV*

>PTEN_isoform_3_206aa
MTAIIKEIVSRNKRRYQEDGFDLDLTYIYPNIIAMGFPAERLEGVYRNNIDDVVSCAERH
YDTAKFNCRVAQYPFEDHNPPQLELIKPFCEDLDQWLSEDDNHVAAIHCKAGKGRTGVMI
CAYLLHRGKFLKAQEALDFYGEVRTRDKKADPTGGIPDKGIIVIGDGSSMDVIAP*

>**sv.4_predicted protein | non-coding**
MTAIIKEIVSRNKRRYQEDGFDLDLTYIYPNIIAMGFPAERLEGVYRNNIDDVVRFLDSK
HKNHYKIYNLCAERHYDTAKFNCRVAQYPFEDHNPPQLELIKPFCEDLDQWLSEDDNHVA
AIHCKAGKGRTGVMICAYLLHRGKFLKAQEALDFYGEVRTRDKKADPTGGIPDKGIIVIG
DGSSMDVIAP*

>**sv.5_predicted protein | non-coding**
MTAIIKEIVSRNKRRYQEDGFDLDLTYIYPNIIAMGFPAERLEGVYRNNIDDVVSCAERH
YDTAKFNCRVAQYPFEDHNPPQLELIKPFCEDLDQWLSEDDNHVAAIHCKAGKGRTGVMI
CAYLLHRGKFLKAQEALDFYGEVRTRDKKADPTGGIPDKGIIVIGDGSSMDVIAP*

>**sv.6_predicted protein | non-coding**
MTAIIKEIVSRNKRRYQEDGFDLDLTYIYPNIIAMGFPAERLEGVYRNNIDDVVRFLDSK
HKNHYKIYNLCAERHYDTAKFNCRVAQYPFEDHNPPQLELIKPFCEDLDQWLSEDDNHVA
AIHCKAGKGRTGVMICAYLLHRGKFLKAQEALDFYGEVRTRDKKILSLWSAS*

>**sv.7_predicted protein | non-coding**
MTAIIKEIVSRNKRRYQEDGFDLDLTYIYPNIIAMGFPAERLEGVYRNNIDDVVRFLDSK
HKNHYKIYNLE*

>**sv.8_predicted protein | non-coding**
MTAIIKEIVSRNKRRYQEDGFDLDLTYIYPNIIAMGFPAERLEGVYRNNIDDVVRE*

>**sv.9_predicted protein | non-coding**
MTAIIKEIVSRNKRRYQEDGFDLDLTYIYPNIIAMGFPAERLEGVYRNNIDDVVRSSVCG
LPAKGEDIFLQFRTHTTGRQVHVL*

>**sv.10_predicted protein | coding**
MTAIIKEIVSRNKRRYQEDGFDLDLTYIYPNIIAMGFPAERLEGVYRNNIDDVVRFLDSK
HKNHYKIYNL*

>**sv.11_predicted protein | non-coding**
MTAIIKEIVSRNKRRYQEDGFDLDLTYIYPNIIAMGFPAERLEGVYRNNIDDVVSCAERH
YDTAKFNCRVAQYPFEDHNPPQLELIKPFCEDLDQWLSEDDNHVAAIHCKAGKGRTGVMI
CAYLLHRGKFLKAQEALDFYGEVRTRDKKILSLWSAS*

>**sv.12_predicted protein | coding**
MTAIIKEIVSRNKRRYQEDGFDLDLTYIYPNIIAMGFPAERLEGVYRNNIDDVVSCAERH
YDTAKFNCRVAQYPFEDHNPPQLELIKPFCEDLDQWLSEDDNHVAAIHCKAGKGRTGVMI
CAYLLHRGKFLKAQEALDFYGEVRTRDKKGVTIPSQRRYVYYYSYLLKNHLDYRPVALLF
HKMMFETIPMFSGGTCNPQFVVCQLKVKIYSSNSGPTRREDKFMYFEFPQPLPVCGDIKV
EFFHKQNKMLKKDKMFHFWVNTFFIPGPEETSEKVENGSLCDQEIDSICSIERADNDKEY
LVLTLTKNDLDKANKDKANRYFSPNFKVKLYFTKTVEEPSNPEASSSTSVTPDVSDNEPD
HYRYSDTTDSDPENEPFDEDQHTQITKV*

>**sv.13_predicted protein | non-coding**
MTAIIKEIVSRNKRRYQEDGFDLDLTYIYPNIIAMGFPAERLEGVYRNNIDDVVRFLDSK
HKNHYKIYNLCTISF*

>**sv.14_predicted protein | non-coding**
MTAIIKEIVSRNKRRYQEDGFDLDLTYIYPNIIAMGFPAERLEGVYRNNIDDVVRIGGSC
PMSLGCFPNEIPKASSPSGFSGC*

>**sv.15_predicted protein | non-coding**
MTAIIKEIVSRNKRRYQEDGFDLDLTYIYPNIIAMGFPAERLEGVYRNNIDDVVRFLDSK
HKNHYKIYNLWGI*

>**sv.16_predicted protein | non-coding**
MTAIIKEIVSRNKRRYQEDGFDLDLTYIYPNIIAMGFPAERLEGVYRNNIDDVVRFLDSK
HKNHYKIYNLWGI*

>**sv.17_predicted protein | non-coding**
MTAIIKEIVSRNKRRYQEDGFDLDLTYIYPNIIAMGFPAERLEGVYRNNIDDVVRFLDSK
HKNHYKIYNLWGI*

>**sv.18_predicted protein | non-coding**
MTAIIKEIVSRNKRRYQEDGFDLDLTYIYPNIIAMGFPAERLEGVYRNNIDDVVRFLDSK
HKNHYKIYNLCAERHYDTAKFNCRVNREVLDSYNSCEKT*

>**sv.19_predicted protein | coding**
MTAIIKEIVSRNKRRYQEDGFDLDLTYIYPNIIAMGFPAERLEGVYRNNIDDVVRFLDSK
HKNHYKIYNLCAERHYDTAKFNCRVAQYPFEDHNPPQLELIKPFCEDLDQWLSEDDNHVA
AIHCKAGKGRTGVMICAYLLHRGKFLKAQEALDFYGEVRTRDKKGVTIPSQRRYVYYYSY
LLKNHLDYRPVALLFHKMMFETIPMFSGGTCNPQFVVCQLKVKIYSSNSGPTRREDKFMY
FEFPQPLPVCGDIKVEFFHKQNKMLKK*

>**sv.20_predicted protein | non-coding**
MTAIIKEIVSRNKRRYQEDGFDLDLTYIYPNIIAMGFPAERLEGVYRNNIDDVVRFLDSK
HKNHYKIYNLCAERHYDTAKFNCRVAQYPFEDHNPPQLELIKPFCEDLDQWLSEDDNHVA
AIHCKAGKGRTGVMICAYLLHRGKFLKAQEALDFYGEVRTRDKKGVTIPSQRRYVYYYSY
LLKNHLDYRPVALLFHKMMFETIPMFSGGTCNPQFVVCQLKVKIYSSNSGPTRREDKFMY
FEFPQPLPVCGDIKVEFFHKQNKMLKKDKMFHFWVNTFFIPGPEETSEKVENGSLCDQEI
DSICSIERADNDKEYLVLTLTKNDLDKANKDKANRYFSPNFKQR*

>**sv.21_predicted protein | coding**
MTAIIKEIVSRNKRRYQEDGFDLDLTYIYPNIIAMGFPAERLEGVYRNNIDDVVRFLDSKH
KNHYKIYNLCAERHYDTAKFNCRGYLFTTVAQYPFEDHNPPQLELIKPFCEDLDQWLSEDD
NHVAAIHCKAGKGRTGVMICAYLLHRGKFLKAQEALDFYGEVRTRDKKGVTIPSQRRYVYY
YSYLLKNHLDYRPVALLFHKMMFETIPMFSGGTCNPQFVVCQLKVKIYSSNSGPTRREDKF
MYFEFPQPLPVCGDIKVEFFHKQNKMLKKDKMFHFWVNTFFIPGPEETSEKVENGSLCDQE
IDSICSIERADNDKEYLVLTLTKNDLDKANKDKANRYFSPNFKVKLYFTKTVEEPSNPEAS
SSTSVTPDVSDNEPDHYRYSDTTDSDPENEPFDEDQHTQITKV*

>**sv.22_predicted protein | coding**
MTAIIKEIVSRNKRRYQEDGFDLDLTYIYPNIIAMGFPAERLEGVYRNNIDDVVSCAERHY
DTAKFNCRGYLFTTVAQYPFEDHNPPQLELIKPFCEDLDQWLSEDDNHVAAIHCKAGKGRT
GVMICAYLLHRGKFLKAQEALDFYGEVRTRDKKGVTIPSQRRYVYYYSYLLKNHLDYRPVA
LLFHKMMFETIPMFSGGTCNPQFVVCQLKVKIYSSNSGPTRREDKFMYFEFPQPLPVCGDI
KVEFFHKQNKMLKKDKMFHFWVNTFFIPGPEETSEKVENGSLCDQEIDSICSIERADNDKE
YLVLTLTKNDLDKANKDKANRYFSPNFKVKLYFTKTVEEPSNPEASSSTSVTPDVSDNEPD
HYRYSDTTDSDPENEPFDEDQHTQITKV*

>**sv.23_predicted protein | coding**
MTAIIKEIVSRNKRRYQEDGFDLDLTYIYPNIIAMGFPAERLEGVYRNNIDDVVRFLDSKH
KNHYKIYNLCAERHYDTAKFNCRVAQYPFEDHNPPQLELIKPFCEDLDQWLSEDDNHVAAI
HCKAGKGRTGVMICAYLLHRGKFLKAQEALDFYGEVRTRDKKADPTGGIPDKGIIVIGDGS
SMDVIAP*

>**sv.24_predicted protein | non-coding**
MTAIIKEIVSRNKRRYQEDGFDLDLTYIYPNIIAMGFPAERLEGVYRNNIDDVVRFLDSKH
KNHYKIYNLCAERHYDTAKFNCRVAQYPFEDHNPPQLELIKPFCEDLDQWLSEDDNHVAAI
HCKAGKGRTGVMICAYLLHRGKFLKAQEALDFYGEVRTRDKKGEEGCVYNF*

>**sv.25_predicted protein | non-coding**
MTAIIKEIVSRNKRRYQEDGFDLDLTYIYPNIIAMGFPAERLEGVYRNNIDDVVRFLDSKH
KNHYKIYNLCAERHYDTAKFNCREDLSCHTKG*

>**sv.26_predicted protein | non-coding**
MTAIIKEIVSRNKRRYQEDGFDLDLTCIHFCGCSSLPFCHSLRTWE*

>**sv.27_predicted protein | non-coding**
MTAIIKEIVSRNKRRYQEDGFDLDLTCIHFCGCSSLPFCHSLRTWE*

Supplementary Fig. (2). *In silico* ORF query results for all the described novel *PTEN* transcripts. Each transcript was predicted as coding mRNA or non-coding RNA, based on the rule of the nonsense-mediated mRNA decay (NMD) pathway.
